# Supplementary material for: Covasim: An agent-based model of COVID-19 dynamics and interventions
Source: PLoS Comput Biol. 2021 Jul 26;17(7):e1009149. doi: 10.1371/journal.pcbi.1009149 (PMC8341708; doi:10.1371/journal.pcbi.1009149)
Supplement: Attachment — Submitted filename: covasim_response_to_reviewers.pdf [file pcbi.1009149.s001.pdf]

Response to reviewers: "Covasim: an agent-based model of COVID-19 dynamics and interventions"

We thank the reviewers for their helpful comments, which we answer in full below. In addition, we would like to flag three other changes that, although not directly requested by the reviewers, were inspired by their comments:

- We have revisited the literature on durations for each disease stage, with many of the values being updated (albeit only slightly, and differences to the results are in most cases less than stochastic and parametric uncertainty). We have updated Table 1 with the new values.
- Following Reviewer 1's comment about adding more information on the most "important" parts of the library, we have merged Figs. 8 and 9 into a new Fig. 8A and 8B, and introduced a new Fig. 9 which includes a second usage example illustrating an additional set of Covasim features.
- Following Reviewer 2's comment about model speed being independent of the number of people infected, we found an additional performance optimization in the integration loop which yielded a significant performance increase.

Reviewer 1

Comment	Response
This paper introduces an agent-based simulation platform specialized on the COVID-19 pandemic. The organization, documentation and testing coverage of the code seems to have production quality, and in terms of performance and features reaches state-of-the-art. The paper is well written. We only have some very minors comments:<br><br>* In our view, the introduction would benefit if it would feature the most "important" points of the library, what set it appart from other agent-base models. What we personally found most interesting is:<br>* The dynamic rescaling feature, which seems to be a very elegant way to solve the computational problem of the interpolation between the low numbers stochastic nature of spreading and the mean-field dynamics dynamics at high case numbers.<br>* The possible integration with Optuna, a modern hyperparameter optimization library, which allows to calibrate the model by comparing its output to case and death numbers. This also allows a straightforward parallelizability of the simulations<br>* C-like performance because of the Numba integration<br>* It's success in predicting the decrease in	Thank you for the very kind comments! We have made the following changes to the manuscript to hopefully describe these features of Covasim in more detail:<br>• In the Introduction: "Overall, the design principle we followed with Covasim was to make common usage patterns as simple as possible, while still giving the user the ability to customize virtually all aspects of the simulation. For example, Covasim comes pre-loaded with demographic data for each country (Section 2.4), but users can also define custom populations and contact networks down to the level of a single city (25) or even university (26). In addition, Covasim includes six built-in interventions (Section 2.5), but custom interventions of arbitrary complexity can also be defined (Fig. 9). In addition, Covasim's high performance for an agent-based model, achieved via dynamic rescaling (Section 2.6.2) and array-based computations (Section 2.7.1), means that most analyses can be run on a standard laptop, removing the need to use a high-performance computing cluster except for large parameter sweeps or model calibrations (Section 2.6.8). These design choices are intended to allow users to start running simple Covasim analyses quickly, while providing flexibility later if more detailed data become available or if the modeling questions become more nuanced." [...] "This paper describes the methodology underlying Covasim, and provides several examples illustrating its use, including an application to Seattle where Covasim scenarios were used to inform a rapid

infections because of the enactment of slight restrictions in the Seattle area.	policy decision, with subsequent validation of these findings by real-world data."<br>• A new figure illustrating additional (even simpler) use cases: "Fig. 9: (A) Illustrative example of a scenario comparison using a simple custom intervention ("protecting the elderly", i.e. removing all transmission among people over age 70 after a certain date). (B) Full listing of the code for this simulation, showing the intervention definition (lines 3–6), and a compact way of creating, running, and plotting the simulations (lines 8–11)."
* In the figure 6, the unit of the y-axis of the cpu-time plot is unclear. It doesn't seem to be cpu-seconds per simulation day as this would be a few orders of magnitude off from the performance example described in the text and the linear increase labelling in the plot.	We have clarified that the simulations are run over 100 days. In addition to updating the y-axis label, we have added the following text: "Covasim performance in terms of processor usage (top) and memory usage (bottom), for the number of agents shown, simulated for 100 days. There is nearly linear scaling over three orders of magnitude of population size." We have used a unit of 100 days since this is relevant to the durations users typically run the model for (initially 60-180 days, unfortunately now 400+!).
* We had the impression, that in the current state, the model is best suited to simulate the propagation on a city level. How well could the model be extended to include another higher level network, for example the propagation between cities, states or countries? Some sort of mobility data is often available which allows a direct modelling of the network without making too much assumptions. If you find this point relevant it would eventually be interesting to touch on it in the discussion.	Indeed, as of now, Covasim has been used to model populations as a single region without more detailed information to extend the networks to include geography and the mobility between different regions like cities, states, or even countries. When we first started developing the model, many locations around the world were showing significantly reduced mobility and encouraging social distancing, so we focused our model development to capture other aspects of COVID-19 transmission instead of mobility. We agree with the reviewer that mobility data could be integrated to model movement of agents to different regions and our team is currently working on this. Part of what makes agent based models both powerful and complex to develop is how to determine which agents will move where and when, especially in the absence of data with these details. To our knowledge many mobility data available indicate the volume change in visitors or traffic to locations (e.g., Google Mobility Trends, SafeGraph). For the kind of network mobility suggested here, we are looking into data on the mobility flows between regions (cell phone based datasets, or even Facebook's Commuting Zones data) to inform where agents are going depending on their home location and the ages of mobile agents. We thank the reviewer for their suggestion and have added the following text to the discussion section on this point: "With the deployment of vaccines come additional questions and interest regarding the lifting of mobility restrictions and social distancing guidelines, as well as questions about

	equitable vaccine distribution to different populations around the world. Additional Covasim development of data driven modeling of mobility will help address the modeling and identification of the risk of importation to regions with fewer resources for early detection and treatment as pre-pandemic mobility gradually returns to parts of the world.”

Reviewer 2

**Comment**	**Response**
This paper presents Covasim which is a comprehensive agent-based model for Covid-19. The paper describes the underlying model and software which is open-source and has been developed by a team from multiple institutions. It does not describe the details of calibration methods or results, which have been presented by the authors and external users of Covasim in other publications (i.e. not in scope for this paper). The model includes all the key aspects for modelling both the dynamics of the virus and disease, as well as interventions to reduce infections. The software is written to a high standard, transparent, and easy to use and extend. A testament to this is that it has already been used by a number of external researchers beyond the core development team and has been used to advice policy makers in multiple countries. The paper is well written, clear and easy to read.<br><br>Specific comments and questions:	Thank you for the positive comments.
1. The default parameters correspond to a doubling time of 4-6 days and and an R0 of 2.2-27. Is the range stochastic uncertainty, if so what is the mean? Assuming the mean of these ranges, the doubling time seems a little high and the R0 seems a little low, certainly when compared to the early stages of the epidemic in Europe.	Since these numbers are so context-specific, we have revised the manuscript to make it clearer that default values should not be used without adjustment: "The value of $\beta = 0.016$ that is currently used as the default in Covasim was initially based on calibrations to data from Washington and Oregon states. However, this default value is too low for high-transmission contexts such as New York City or Lombardy (48), and may be too high for low-transmission contexts such as India (49). Hence, this parameter must be calibrated by the user to match local epidemic data, as described in Section 2.6.8."<br><br>We have also tried to clarify the language indicating that the ranges are *due* to the factors mentioned: "For a well-mixed

<table>
<tr><td></td><td>population where each individual has an average of 20 contacts per day, a value of β = 0.016 corresponds to a doubling time of roughly 4–6 days and an R0 of approximately 2.2–2.7, with the exact value depending on the population size, age structure, and other factors."</td></tr>
<tr><td>2. Fig 2 - Viral load timing. It seems that out of those who do go on to develop symptoms, their viral load (and thus infectiousness) will be zero prior to the onset of symptoms or non-zero for at most one day. What is the breakdown in transmission by symptom status of the source? Is the epidemic predominantly being driven by transmission from symptomatic individuals? What is the mean generation time?</td><td>It is true that in the figure shown, viral load rises at most one day before symptoms. However, in the model, individuals can become infectious more than five days prior to showing symptoms. This is shown in Fig. 2D of the companion paper, reproduced here:
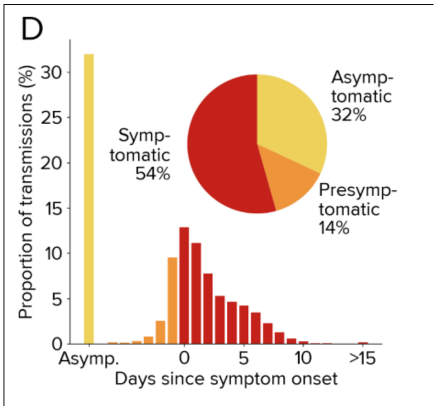

We have regenerated Fig. 2 using a different random seed to include a case where viral load rises more than one day before symptoms, and added a citation to the aforementioned preprint: "Viral loads for a representative sample of individuals given default parameter values are shown in Fig. 2. The proportion of transmissions by asymptomatic, presymptomatic, and symptomatic individuals varies by context; estimated proportions for Seattle are shown in (25)."</td></tr>
<tr><td>3. Fig 3 - not really necessary, all the information is in Fig 4 (which is very clear).</td><td>We (the authors) had a lively debate regarding this figure, and found there were a variety of opinions regarding its value (as well as the clarity of Fig. 4, though we appreciate the reviewer's comment). Given the difficulty of explaining and representing network connectivity, we did not want to remove Fig. 3 for the subset of readers who may find it helpful. Instead, we have added a second panel to it, which we hope bridges between the abstract-but-intuitive representation in (now) Fig. 3A and the concrete-but-unintuitive representation in Fig. 4. The new Fig. 3B uses data from the model, as in Fig. 4, but shows connections across layers for individuals, as in Fig. 3A; unlike either Fig. 3A or Fig. 4, it also shows default weights for each connection. We appreciate that there may be relatively few readers who get value out of all of Fig. 3A, Fig. 3B, and Fig. 4; however, we wish to keep all three to communicate with the broadest readership possible.</td></tr>
<tr><td>4. Contact tracing - what proportion of</td><td>Most of these are context-dependent and can (and should) be</td></tr>
</table>

interactions are typically contact-traced? Does this depend upon the number of new infections (i.e. during very high incidence contract-tracing teams can get saturated)? How many days of prior interactions are traced? Have you modelled digital contract tracing, such as the Google-Apple Exposure Notification System?	informed by local data. To illustrate this, we have updated Fig. 11 to include a new panel showing the calibration to the reported number of contacts traced; a full explanation of the methodology is given in the companion paper.<br><br>We have also added the following text to the section on contact tracing: "Digital contact tracing can be approximated in Covasim as a standard contact-tracing intervention with zero delays, with the caveat that tracing multiple steps (i.e., contacts of contacts) within a single day would require a custom intervention." As the reviewer is no doubt aware, digital contact tracing has been implemented in much greater detail in other models (e.g. Ferretti et al., Hinch et al.). These papers are both already cited in the manuscript.
5. Isolation/Quarantine - please can you give more details of how the probability of transmission is lowered. Is this done by changing the contact network (i.e. 80% of quarantined stay at home so only interact on their household network) or by a change in the per-interaction infectious rate? (or a mix of both)	We have clarified this: "For performance reasons, isolation and quarantine are implemented as reductions in per-contact transmission risk rather than changes in the number of contacts; for realistic parameter values (i.e., $\beta \ll 1$), the difference between these implementations should be negligible."
6. Performance - considering the model is written in pure Python it has incredibly impressive performance, which is due to clever coding and the use of efficient packages such as Numba. What (if any) limitations does the array approach have compared to a more traditional object-oriented approach? Is performance dependent on the number of people infected and the interventions? If so, what are the conditions in the Fig 6. The text mentions that you use Sciris for parallelization, is the reported performance for a single processor and single thread, or are multiple processors and multi-threading being used?	We also thank the reviewer for bringing our attention the issue regarding uninfected people. Although the speed does not depend on the number of infected agents, this comment prompted us to ask why not, and hence rethink the logic of the innermost loop. To our surprise, a change in array indexing resulted in a significant overall speed increase. This change, along with other updates, has resulted in a 3.7-fold speed increase in the latest version of Covasim (v2.1.1) compared to when benchmarking was originally performed (v1.7.0). Fig. 6 has been updated to reflect the new results. To test this result locally, run:<br><br>`import covasim as cv`<br>`sim = cv.Sim(pop_size=100e3, n_days=100)`<br>`sim.initialize()`<br>`sim.run()`<br><br>This simulation, for 10 million person-days, should take about 1.2-1.5 s to run for version 2.1.1.<br><br>We have also clarified the text in several places: "Performance scales linearly with population size [...] single-core compute time [...] scales at a rate of roughly 7 million simulated person-days per second of CPU time. [...] One consequence of the array-based implementation is that compute time depends on the number of agents and the number of connections per agent, but is independent of the number of infected agents; this is because uninfected agents are simply represented as zeros in the transmission probabilities vector.

	[...] Covasim [...] can also be adapted easily to other parallel processing libraries such as Celery and Dask. Although in some special situations it is possible to split a single simulation across multiple cores, parallel processing is used primarily to run multiple independent simulations simultaneously, such as for uncertainty analyses or calibration."
7. Deployment - it is mentioned that it can be run in R using 'reticulate' (as can most Python code). Do you have a wrapper for Covasim in R using reticulate? Have you thought putting Covasim on CRAN or Bioconductor?	We do not have a full R wrapper, though we do include a reticulate usage example in the repository, and cover it in the FAQ. Our team unfortunately does not have much expertise in R, so we would welcome external contributions that would improve the experience for R users. Perhaps surprisingly given R's popularity, we have gotten relatively few questions about using Covasim via R (perhaps because R users do not know it is possible). In future, we may produce Shiny apps based on Covasim, and making that code open source could then serve as a template for other users wishing to use Covasim via R. (We know it is possible since we have made a Shiny app using a Python-based polio model that was adapted from Covasim, but neither this app nor the polio model are ready to be made public.)
8. Software tests - please can you give more details of the tests (and examples).	We have added the following text to that section: "The test suite includes unit tests (e.g., checking that sampling functions produce the desired distributions; that simulations loaded from file exactly match the original), functional tests (e.g., that a simulation run with a particular analyzer produces a plot), and end-to-end "scientific" tests (e.g., that an increase in mortality rate leads to more deaths, while adding NPIs leads to fewer)."
9. Example usage - the example in Fig 9 is very impressive and demonstrates how simple/intuitive the code is for a complex simulation. Is it possible to have a dynamic intervention policy? Interventions such as lockdowns and school closure tend to be timed based on the prevailing incidence or hospital occupancy. Can this be modelled as opposed to interventions set between specific dates?	This is a very good suggestion, and it is possible, although it is not as easy to do currently as it should be. Currently, it can only be done via custom interventions, and there is a (complex) example of it in the code for our "Stepping Back to School" report. We have added the following text to this section: "Each intervention has full access to the simulation object at each timestep, which means that user-defined interventions can dynamically modify any aspect of the simulation. This can be used to create interventions more specific than those included by default in Covasim, such as age-specific physical distancing or quarantine, or interventions that are dynamically "triggered" based on the current or past state of the simulation." We are currently working on implementing a more natural way of doing this.
10. Case study - is a nice example which has been presented in a prior publication. What is the basis of the forecast interval? Is this the stochastic uncertainty of the model in a finite	It is not a rigorous Bayesian credible interval, since it is a combination of stochastic and parametric uncertainty, and since the likelihood function is not well defined. We have clarified this in the manuscript in several places:

size population? Is it a Bayesian interval from uncertainty in the calibration of the model parameters?	• Section 2.6.8: "Intuitively, most distributional assumptions mean that larger errors imply a lower log-likelihood. However, we do not make explicit distributional assumptions, so caution is advised with treating them as statistically rigorous likelihoods."<br>• Section 3.1: "Since these forecast intervals are typically produced by a combination of both stochastic variability ("aleatory uncertainty") and imperfect knowledge of the "true" parameter values ("epistemic uncertainty"), they should not be interpreted as statistically rigorous Bayesian credible intervals (80,81)."<br>• Section 3.3: "We then ran the model with eight different calibrated model parameter sets (with multiple parameter sets used to capture parametric uncertainty) to (a) estimate unobserved quantities [...] (the large uncertainty interval for deaths is a consequence of the small numbers of events being predicted, i.e., fewer than 10 deaths per day; this forecast interval includes both parametric and stochastic uncertainty, as described in Section 3.1)".
